# Hepatitis B virus X protein-induced upregulation of CAT-1 stimulates proliferation and inhibits apoptosis in hepatocellular carcinoma cells

**DOI:** 10.18632/oncotarget.17631

**Published:** 2017-05-05

**Authors:** Rongjuan Dai, Feng Peng, Xinqiang Xiao, Xing Gong, Yongfang Jiang, Min Zhang, Yi Tian, Yun Xu, Jing Ma, Mingming Li, Yue Luo, Guozhong Gong

**Affiliations:** ^1^ Department of Infectious Diseases, Institute of Hepatology Central South University, Second Xiangya Hospital, Central South University, Changsha, Hunan 410011, PR China

**Keywords:** HBx, CAT-1, miR-122, Gld2, HCC

## Abstract

The HBx protein of hepatitis B virus (HBV) is widely recognized to be a critical oncoprotein contributing to the development of HBV-related hepatocellular carcinoma (HCC). In addition, cationic amino acid transporter 1 (CAT-1) gene is a target of miR-122. In this study, we found that CAT-1 protein levels were higher in HBV-related HCC carcinomatous tissues than in para-cancerous tumor tissues, and that CAT-1 promoted HCC cell growth, proliferation, and metastasis. Moreover, HBx-induced decreases in Gld2 and miR-122 levels that contributed to the upregulation of CAT-1 in HCC. These results indicate that a Gld2/miR-122/CAT-1 pathway regulated by HBx likely participates in HBV-related hepatocellular carcinogenesis.

## INTRODUCTION

Hepatitis B virus (HBV) infection is widely recognized as a leading cause of hepatocellular carcinoma (HCC) [1–4], and the HBV x protein (HBx) is crucial to the induction of HBV-related HCC. HBx promotes the formation and development of HCC via many mechanisms, including trans-activation of various host cell genes related to growth and apoptosis, interactions with p53 and the Ras-Raf-MAP kinase pathway, and inhibition of DNA repair [5–7]. Levels of miR-122, which is the most enriched microRNA in normal liver tissues, are markedly decreased in HCC, particularly in HBV-related HCC [8–10]. In a previous study, we found that HBx down-regulated miR-122 in hepatocellular tissue by reducing Germ Line Development 2 (Gld2) protein levels [11]. Whether HBx-induced downregulation of miR-122 promotes the formation and development of HBV-related HCC remains unknown.

The CAT-1 protein, which is encoded by the Solute Carrier Family 7 Member 1 (SLC7A1) gene, transports cationic amino acids and is a confirmed target gene of miR-122 [12]. Previous studies have demonstrated that CAT-1 expression is highest in the embryonic liver; expression decreases by about 70% at birth and is almost nonexistent in adulthood [8, 13, 14]. L-Arginine, which is transported into cells by CAT-1, is a precursor of polyamines and nitric oxide (NO), both of which help regulate cell differentiation and proliferation [15, 16]. Since arginine is necessary for the survival of human hepatoma cells [15, 17], CAT-1 might also contribute to HCC progression.

In this study, we examined miR-122 and CAT-1 expression, the regulation of CAT-1 by HBx, and the role of CAT-1 in cell growth and proliferation in HBV-related HCC tissues.

## RESULTS

### Expression of miR-122, Gld2, and CAT-1 in HCC tissues and hepatic cell lines

**Figure 1:**
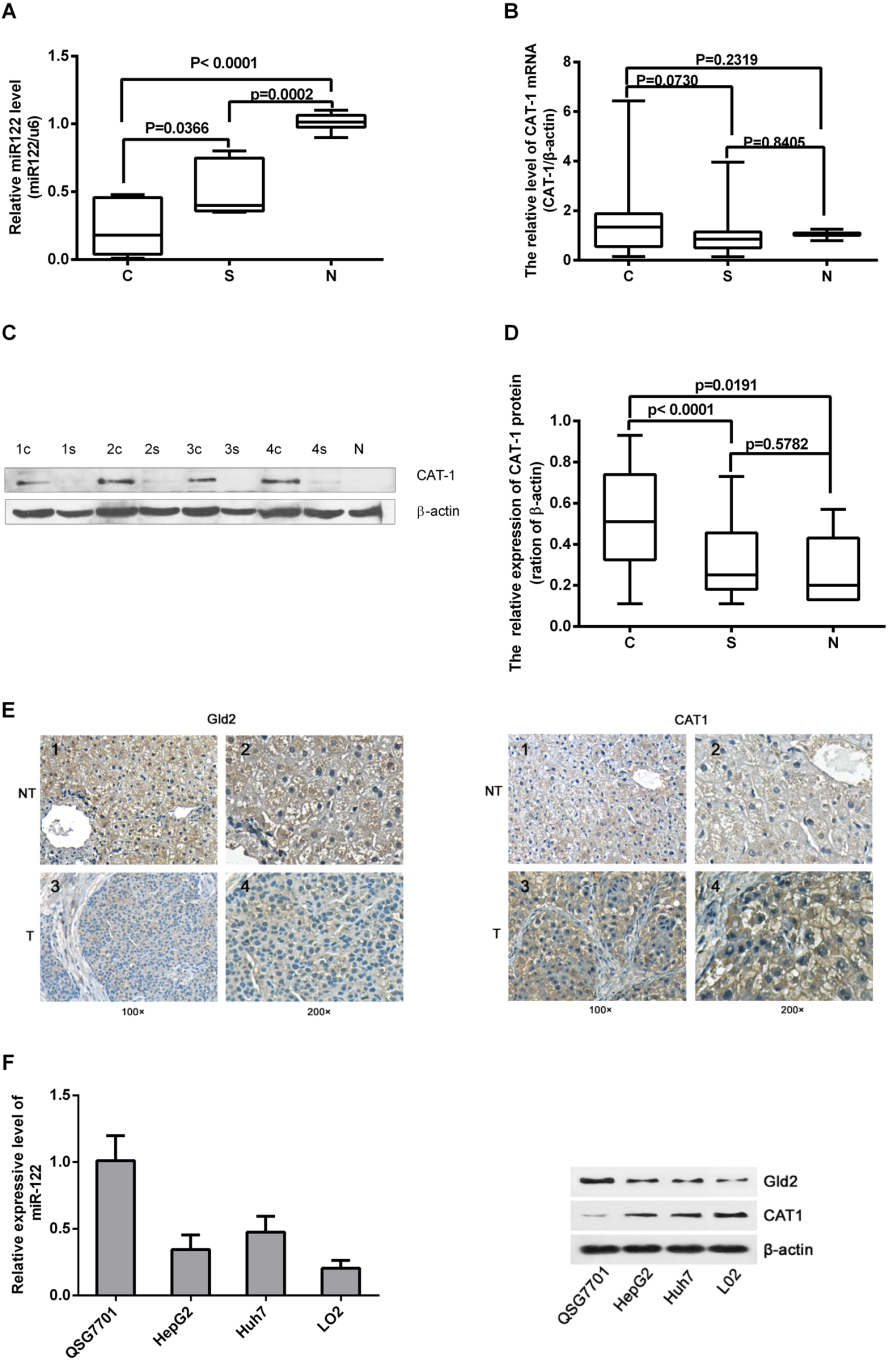
Expression of miR-122, Gld2, and CAT-1 in HCC tissues and hepatic cell lines **(A)** miR-122 level in 30 pairs of HCC versus normal tissue detected by qRT-PCR; miR-122 has significantly lower expression in HCC tissues (c) compared to para-carcinoma tissue (s) (*p*<0.05) and normal tissue (N) (*p*<0.0001). **(B)** Expression of CAT-1 mRNA does not differ significantly among tissues as detected by qRT-PCR (*P*>0.05). **(C, D)** CAT-1 protein levels detected by western blot are significantly higher in HCC tissue than in para-cancerous (*P*<0.0001) or normal tissue (*P*<0.05); no statistically significant difference observed between para-cancerous and normal tissues (*P*>0.05). **(E)** Immunohistochemistry results regarding Gld2 and CAT-1 protein expression in HCC tissue and paired cancer-adjacent tissue. **(F)** RT-qPCR assay of miR-122 in different cell lines. Western blotting utilized to analyze Gld2 and CAT-1 in different cell lines with β-actin as internal reference. Data represent mean ± SD. Experiments were performed in triplicate.

We assessed miR-122 and CAT-1 mRNA and protein levels in 30 paired HCC and para-cancerous tissues and in normal liver tissues via RT-qPCR and Western blot analysis. Relative miR-122 expression was lower in HCC tissues than in para-cancerous tissues or normal tissues (Figure 1A). CAT-1 protein levels were higher in HCC tissues than in para-cancerous or normal tissues (Figure 1C, 1D); however, CAT-1 mRNA levels did not differ among the different types of tissues (Figure 1B). IHC array results also indicated that Gld2 protein levels were reduced and CAT-1 expression was increased in HCC tissues (Figure 1E).

RT-qPCR and Western blot analysis were performed to detect levels of miR-122, Gld2, and CAT-1 in different liver cell lines. MiR-122 and Gld2 protein levels were highest, and CAT-1 protein levels were lowest, in the normal hepatic cell line (QSG7701) (Figure 1F). Compared to QSG7701 cells, miR-122 and Gld2 levels were lower, while CAT-1 levels were higher, in the Huh7 and HepG2 HCC cell lines and in the normal fetal liver L02 cell line (Figure 1F).

### CAT-1 promotes, and CAT-1 knockdown reduces, cell proliferation and increases colony formation

**Figure 2:**
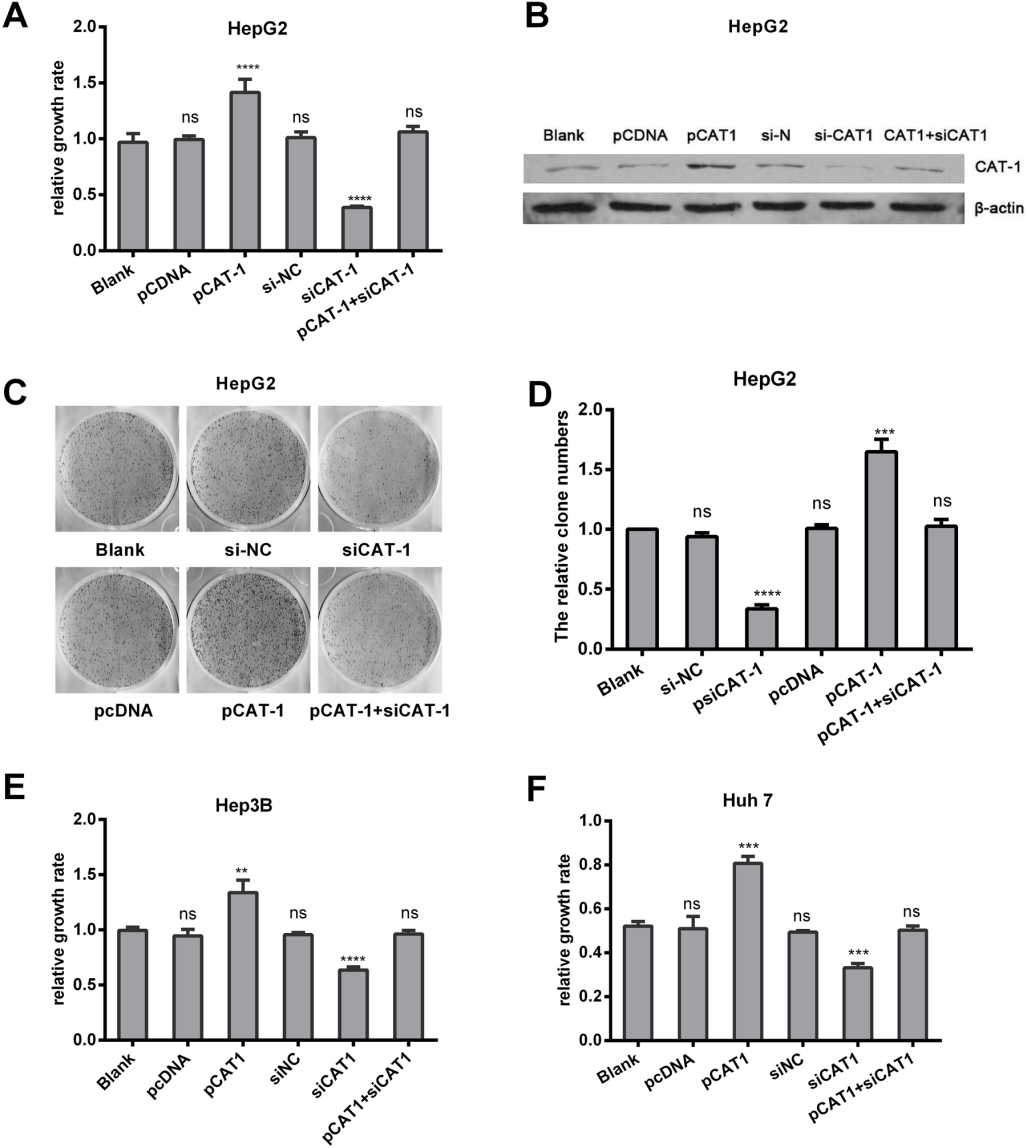
CAT-1 promotes, and CAT-1 knockdown reduces, cell proliferation and increases colony formation **(A)** Cell proliferation of HepG2 transfected with pCAT-1, pcDNA, psiCAT-1, psi-NC, pCAT-1 with psiCAT-1 as detected by MTT assay. **(B)** CAT-1 expression analyzed by western blotting with β-actin as internal reference. **(C, D)** Cloning efficiency assessed by cell colony formation assay. **(E)** Cell proliferation of Hep3B transfected with pCAT-1, pcDNA, psiCAT-1, psi-NC, pCAT-1 with psiCAT-1 as-detected by MTT assay. **(F)** Cell proliferation of Huh7 transfected with pCAT-1, pcDNA, psiCAT-1, psi-NC, pCAT-1 with psiCAT-1 as-detected by MTT assay. Statistically significant differences are indicated: *p <0.05, **p<0.01, ***p<0.001, ****p<0.0001. Data represent mean ± SD. Experiments were performed in triplicate.

Since CAT-1 levels were upregulated in HCC, we examined the effects of CAT-1 overexpression and inhibition on hepatoma cell viability. The pCAT-1, pcDNA, psiCAT-1, psi-NC, and pCAT-1 with psiCAT-1 plasmids were transfected into HepG2 cells; non-transfected HepG2 cells served as a negative control. Relative growth rates measured using MTT assays were as follows: control group, 1.00±0.01; pcDNA, 1.00±0.01; pCAT-1, 1.52±0.05; psi-NC, 1.01±0.00; psiCAT-1, 0.37±0.00; and pCAT-1 with psiCAT-1, 1.01±0.01. These results suggest that CAT-1 overexpression promoted, while CAT-1 knockdown reduced, HepG2 cell proliferation (Figure 2A). Similar results were obtained in Hep3B and Huh7 cells (Figure 2E, 2F). Cell colony formation assays were performed in same groups, and the average cloning efficiencies were as follows: control group, 1.00±0.00; pcDNA, 1.01±0.30; pCAT-1, 1.65±0.10; psi-NC, 0.94±0.03; psiCAT-1, 0.34±0.035; and pCAT-1 with psiCAT-1, 1.03±0.06 (Figure 2C, 2D). Successful reduction of CAT-1 protein levels in the siRNA group compared to the controls was confirmed with Western blots (Figure 2B).

### CAT-1 siRNA induces HCC cell apoptosis

**Figure 3:**
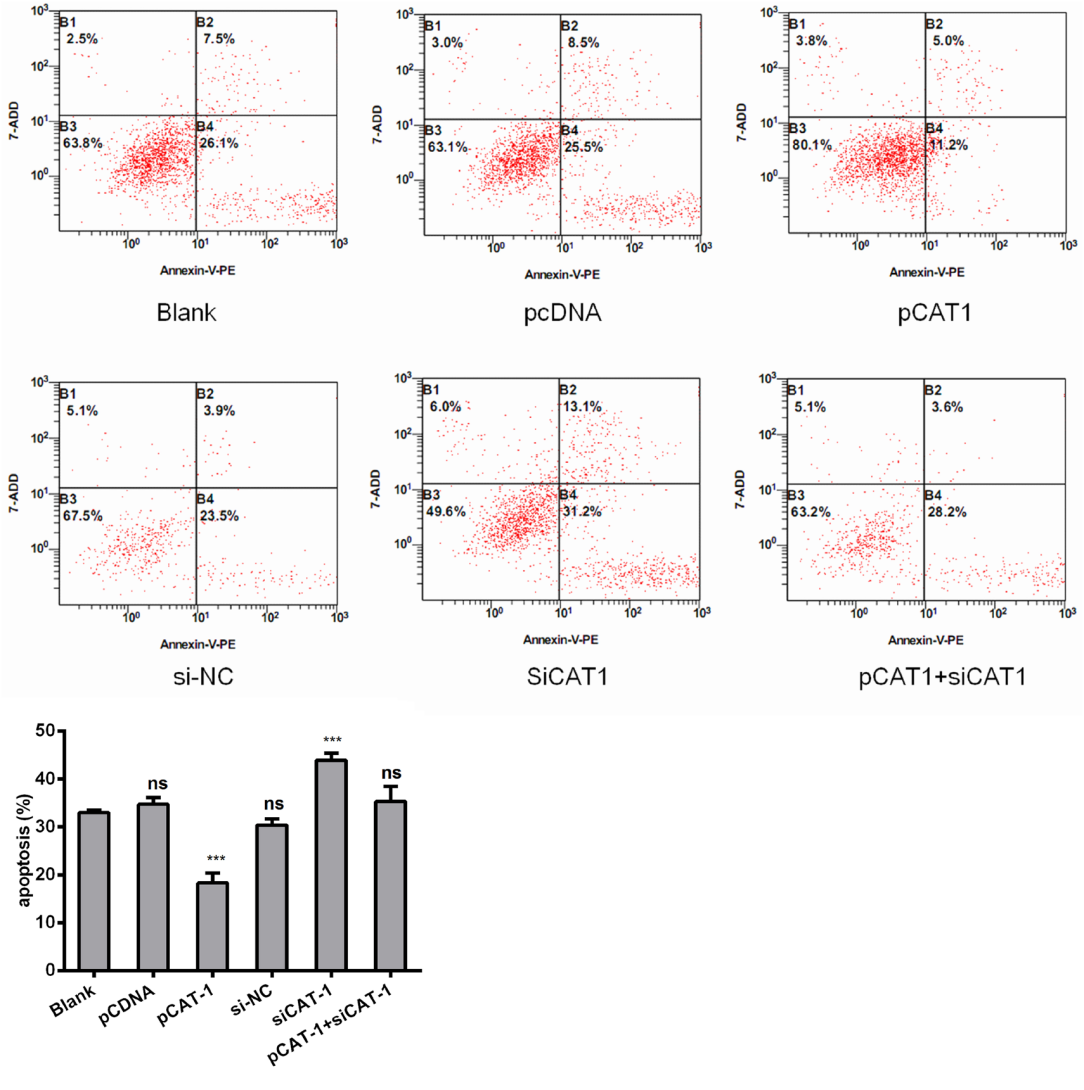
CAT-1 siRNA induces HCC cell apoptosis Flow cytometric analysis utilized to detect apoptosis after transfecting pCAT-1, psiCAT-1, pcDNA, psi-NC, pCAT-1 with psiCAT-1 into HepG2 cells. Statistically significant differences are indicated: *p<0.05, ***p<0.001. Data represent mean ± SD. Experiments were performed in triplicate.

To further examine the effects of CAT-1 knockdown on cell apoptosis, we analyzed HepG2 cells 72 hours after transfection with pCAT-1, pcDNA with psiCAT-1, psi-NC, or pCAT-1 with psiCAT-1 using flow cytometry. After staining with Annexin V-APC/7-AAD probe, apoptotic cells were quantified. CAT-1 overexpression reduced the number of apoptotic cells compared to the blank groups. In contrast, CAT-1 knockdown increased the number of apoptotic cells and reversed the effect of CAT-1 overexpression on HepG2 cell apoptosis (Figure 3, Table 1).

**Table 1:** CAT-1 siRNA induces HCC cell apoptosis

	The ration of apoptosis	P
Blank	33.0±0.57	
pCDNA	34.7±1.48	0.131
pCAT-1	19.6±3.12	0.002
si-NC	28.7±1.19	0.005
siCAT-1	41.2±3.71	0.006
pCAT-1+ siCAT-1	33.7±2.27	0.632

Flow cytometric analysis was applied to detected apoptosis after transfected the pCAT-1, siCAT-1, pCDNA, si-NC, pCAT-1 with siCAT-1 into HepG2 cells. Each group compared to blank groups, statistically significant differences are indicated: p<0.05. We performed triplicate experiments. Data are expressed as mean values±SD.

### CAT-1 increases HCC cell invasive ability

**Figure 4:**
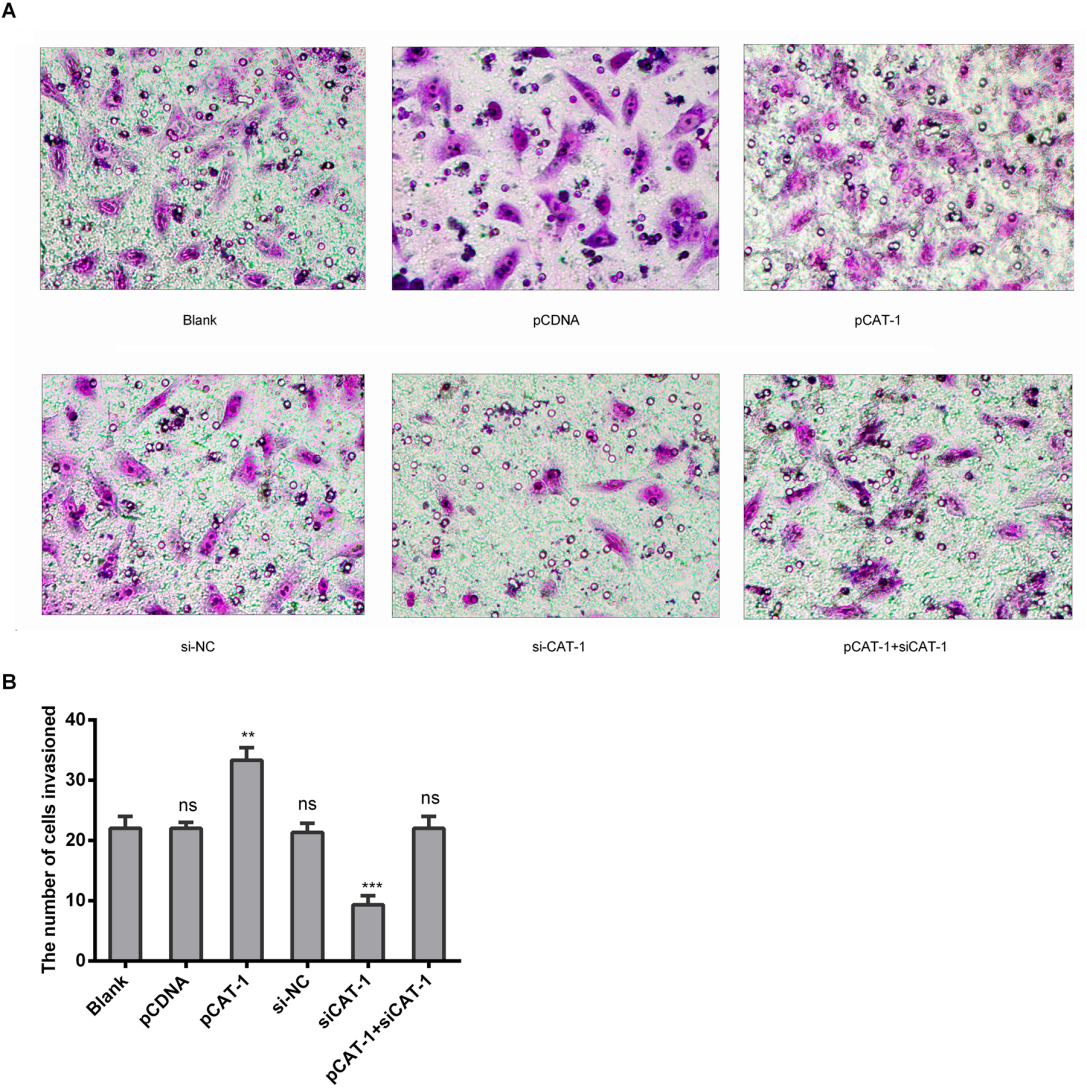
CAT-1 increases HCC cell invasive ability **(A, B)** Metastasis and invasive ability of HepG2 cells treated with pCAT-1, psiCAT-1, pcDNA, psi-NC, pCAT-1 with psiCAT-1 as-determined by Transwell assays. Statistically significant differences are indicated: **p<0.01, ***p<0.001. Data represent mean ± SD. Experiments were performed in triplicate.

To determine whether CAT-1 affects hepatoma cell metastasis, we examined metastasis and invasive ability in HepG2 cells transfected with pCAT-1, pcDNA and psiCAT-1, psi-NC, or pCAT-1 with psiCAT-1 using Transwell assays. CAT-1 overexpression increased, while CAT-1 knockdown inhibited, cell invasion compared to the control group (Figure 4A, 4B).

### HBx promotes inhibits miR-122 to upregulate CAT-1

**Figure 5:**
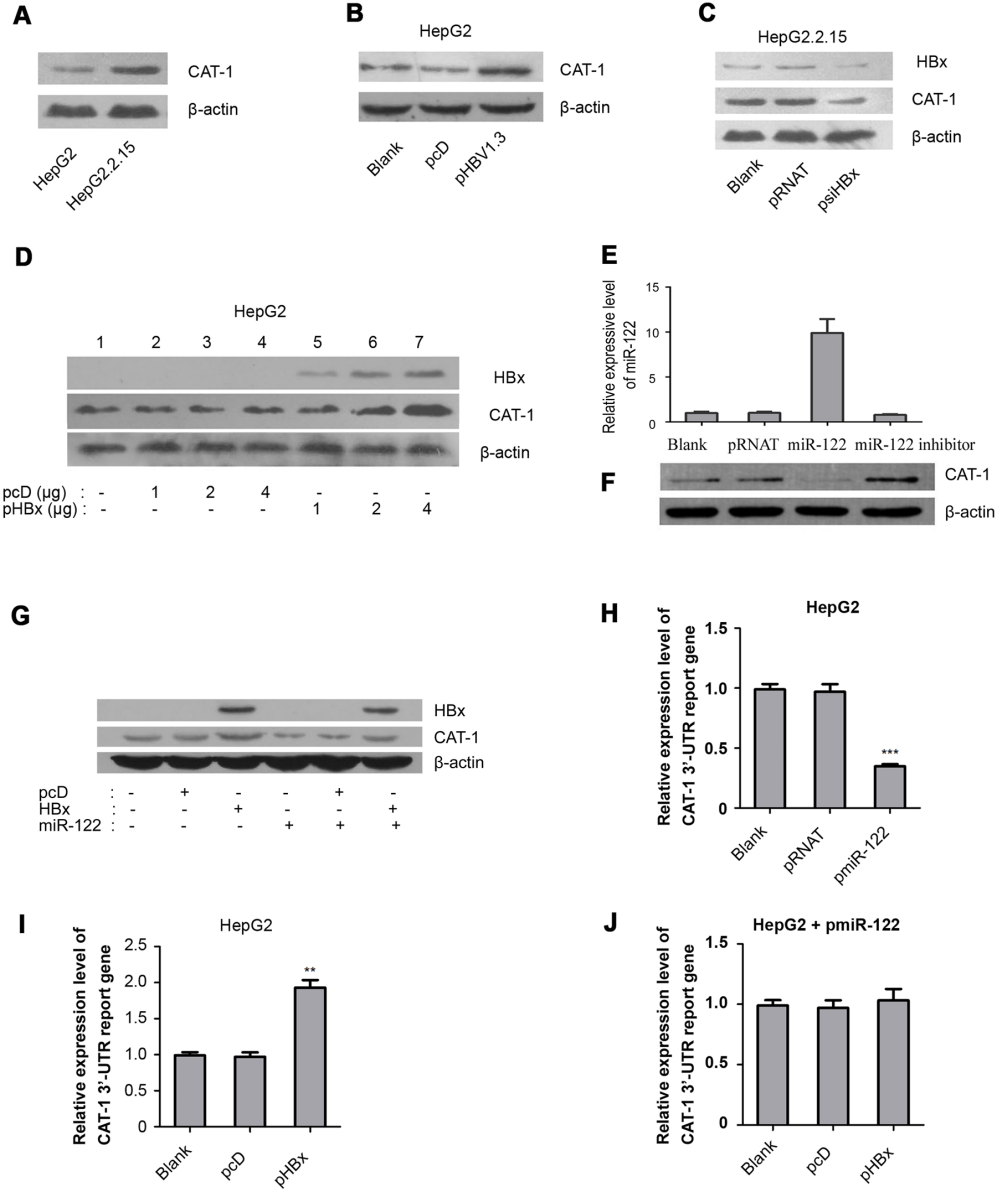
HBx promotes inhibits miR-122 to upregulate CAT-1 **(A)** Western blotting analysis of CAT-1 protein in HepG2 and HepG2.2.15 cells with β-actin as internal reference. **(B)** Western blotting analysis of CAT-1 protein in HepG2 cells transfected with pHBV1.3 and Blank groups with β-actin as internal reference. **(C)** Western blotting analysis of CAT-1 protein in HepG2.2.15 cells transfected with pRNAT or psiHBx and Blank group. **(D)** Western blotting analysis of CAT-1 protein in HepG2 cells transfected HBx-expressing vector in different dosage and Blank groups. **(E)** RT-qPCR assay of miR-122 and **(F)** Western blotting analysis of CAT-1 protein in HepG2 cells transfected with miR-122-expressing vector or miR-122 inhibitor and the Blank groups with β-actin as internal reference. **(G)** Western blotting analysis of CAT-1 protein in miR-122 over-expressing HepG2 cells transfected with HBx-expressing vector or HBx alone without miR-122 and Blank groups. **(H)** Dual-luciferase assay of CAT-1 3’-UTR report gene in miR-122 over-expressing HepG2 cells and Blank groups. **(I)** Dual-luciferase assay of CAT-1 3’-UTR report gene in HepG2 cells transfected with HBx-expressing vector and Blank groups. **(J)** Dual-luciferase assay of CAT-1 3’-UTR report gene in miR-122 over-expressing HepG2 cells transfected with HBx-expressing vector and Blank groups. Statistically significant differences are indicated: * *p*<0.05, ** *p*<0.01, *** *p*<0.001. Data represent mean ± SD. Experiments were performed in triplicate.

Western blot analysis was performed to evaluate the effect of HBV on CAT-1 protein levels in HepG2 and HepG2.2.15 cells. CAT-1 protein levels were higher in HepG2.2.15 cells than in HepG2 cells (Figure 5A). CAT-1 protein levels were also higher in HepG2 cells transfected with pHBV1.3 for 72 hours than in the pcDNA-transfected control group (Figure 5B). These results indicate that HBV increases CAT-1 expression in our *in vitro* model of HBV infection.

To determine whether CAT-1 up-regulation in HBV-infected cells was related to HBx, HepG2.2.15 cells were transfected with psiHBx to inhibit HBx expression; psi-NC was transfected as a control. Western blots indicated that HBx knockdown decreased CAT-1 levels in HBV-infected cells (Figure 5C). We also transfected HepG2 cells with different doses of pHBx for 72 h; HBx increased CAT-1 protein levels in a dose-dependent manner (Figure 5D). Together, these results indicate that HBx contributes to the up-regulation of CAT-1 in HBV-infected hepatic cells.

MiR-122 can also reduce CAT-1 protein levels at the post-transcriptional level, and our previous research demonstrated that HBx down-regulates miR-122 expression in hepatic cells [11]. We therefore examined whether reductions in miR-122 levels contributed to HBx-induced up-regulation of CAT-1.

First, we confirmed that miR-122 reduced CAT-1 protein levels. HepG2 cells were transiently transfected with miR-122 overexpression or miR-122 inhibitor plasmids for 72 h, with psi-NC transfections serving as a control (Figure 5E). Western blots indicated that miR-122 overexpression reduced, while miR-122 knockdown increased, CAT-1 protein levels in hepatic cells (Figure 5F). We next tested whether miR-122 overexpression reversed HBx-induced increases in CAT-1 levels by transiently transfecting HepG2 cells with both pHBx and pcDNA as well as miR-122 overexpression plasmid, pcDNA, or HBx alone without miR-122 as a control; after 72 h, Western blots were used to examine the effects of HBx on CAT-1 levels. Indeed, miR-122 overexpression reversed the HBx-induced increase in CAT-1 levels in HepG2 cells (Figure 5G). Taken together, these observations imply that reduced miR-122 levels likely contribute to HBx-induced increases in CAT-1 levels.

Because miR-122 regulates CAT-1 expression by binding to the CAT-1 mRNA 3'-UTR, we examined whether HBx interacted with the CAT-1 mRNA 3'-UTR in hepatic cells using a dual-luciferase reporter gene assay. First, we tested interactions between miR-122 and the CAT-1 gene by co-transfecting Hep2G cells with a CAT-1 mRNA 3'-UTR reporter vector and either pmiR-122 or psi-NC (control). MiR-122 overexpression inhibited the expression of the CAT-1 mRNA 3'-UTR reporter gene (Figure 5H). Similarly, Hep2G cells were co-transfected with the CAT-1 mRNA 3'-UTR reporter vector and either pHBx or pcDNA (control) to determine whether HBx increased the expression of the CAT-1 mRNA 3'-UTR reporter gene (Figure 5I). In addition, we evaluated the effects of HBx on the CAT-1 mRNA 3'-UTR reporter in miR-122-overexpressing HepG2 cells using a dual-luciferase array to investigate whether miR-122 overexpression could interrupt this pathway. MiR-122 overexpression reversed the effects of HBx on the CAT-1 mRNA 3'-UTR reporter in HepG2 cells (Figure 5J). Together, these results indicate that miR-122 is a critical component of HBx-induced up-regulation of CAT-1.

### CAT-1 siRNA inhibits the tumorigenic effects of HBx in HepG2/HepG2.2.15 cells

**Figure 6:**
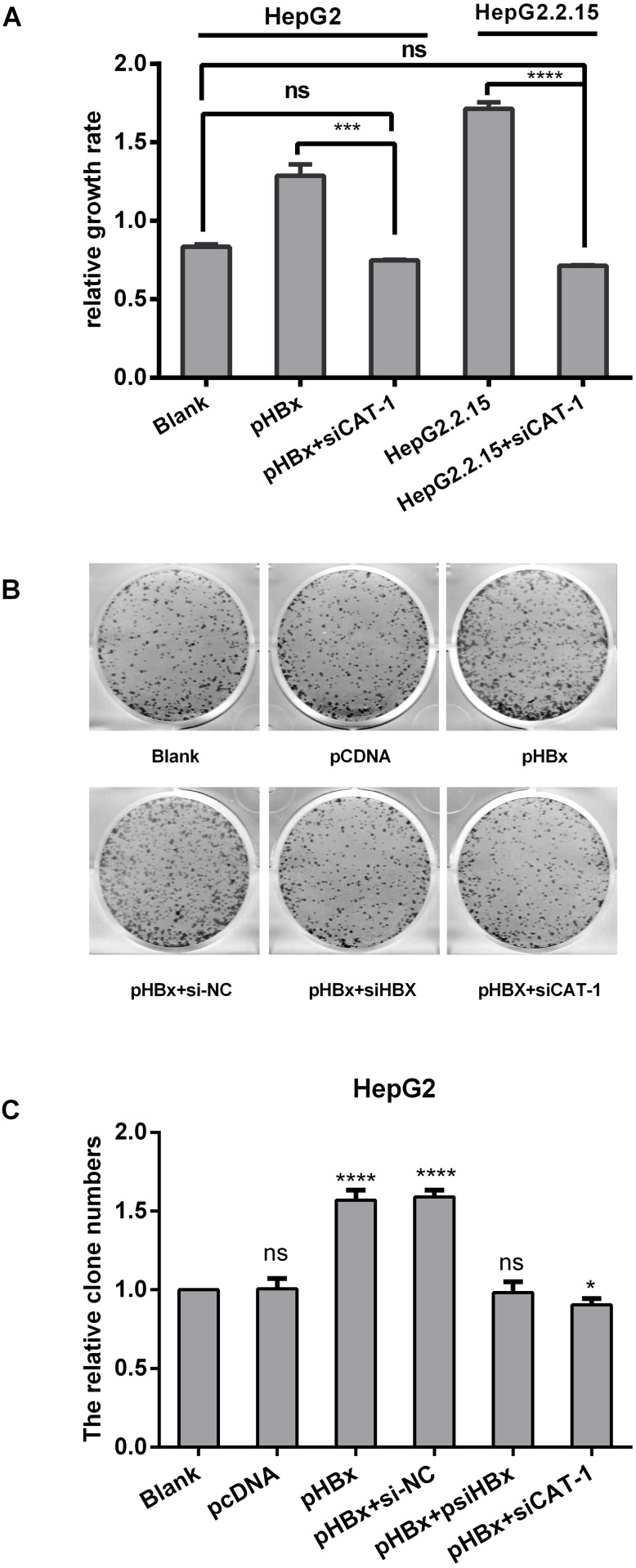
CAT-1 siRNA inhibits the tumorigenic effects of HBx in HepG2/HepG2. 2.15 cells **(A)** Cell proliferation of HepG2 transfected with pHBx, pHBx with psiCAT-1, HepG2.2.15 transfected with psiCAT-1 as-detected by MTT assay. **(B, C)** Clone efficiency of HepG2 transfected with pCDNA, pHBx, pHBx with psi-NC, pHBx with psi-HBx, pHBx with psi-CAT-1 as-assessed by cell colony formation assay. Statistically significant differences are indicated: *p<0.05, ***p<0.001, ****p<0.0001. Data represent mean ± SD. Experiments were performed in triplicate.

In a previous study, we found that HBx up-regulated CAT-1 expression by reducing miR-122 levels and promoting proliferation in HCC cells. Here, we investigated whether siCAT-1 reduced proliferation in HBx-overexpressing HepG2 cells and in HBV-infected HepG2.2.15 cells using MTT and plate colony formation assays. HepG2 and Hep2.2.15 cells were transfected with pHBx, psiCAT-1, or pHBx with psiCAT-1 for 48 h. As shown in Figure 6A, CAT-1 up-regulation increased HBx-induced proliferation, while CAT-1 knockdown reduced proliferation in HBx-expressing cells. HBx overexpression also increased, while siRNA-mediated HBx knockdown decreased, colony formation; siRNA-induced CAT-1 knockdown in the presence of HBx similarly decreased colony formation (Figure 6B, 6C). Together, these results indicate that CAT-1 knockdown can reverse HBx-induced increases in HCC survival.

### CAT-1 siRNA suppresses invasive ability in the presence of HBx in hepatoma cells

**Figure 7:**
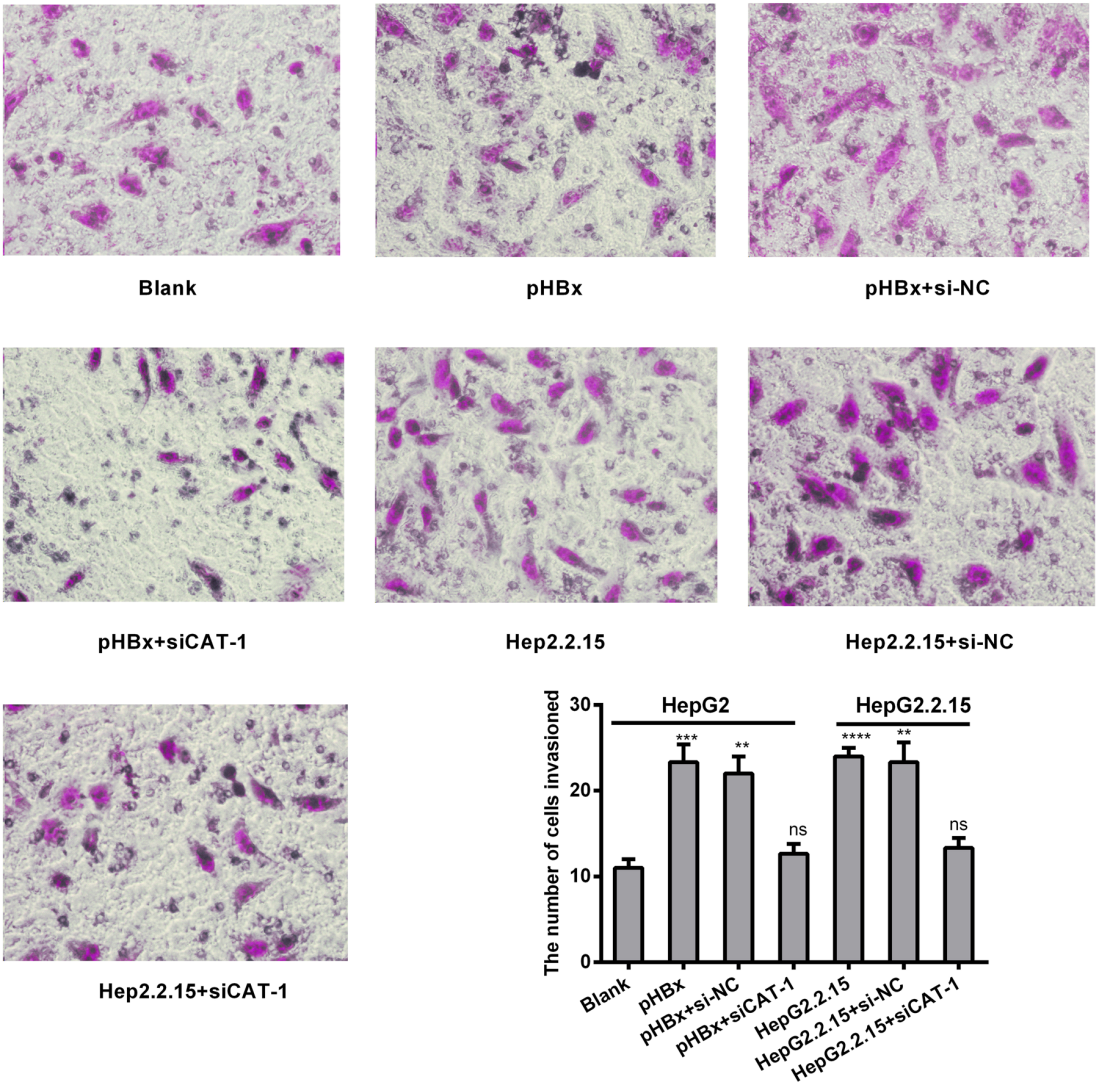
CAT-1 siRNA suppresses invasive ability in the presence of HBx in hepatoma cells **(A, B)** Invasive ability of HepG2 transfected with pHBx, pHBx with psi-NC, pHBx with psiCAT-1, HepG2.2.15 transfected with psi-NC, psiCAT-1 as-assessed by invasion assay. Statistically significant differences are indicated: **p <0.01, ***p<0.001, ****p<0.0001. Data represent mean ± SD. Experiments were performed in triplicate.

We next investigated the effects of HBx and CAT-1 on cell invasion using a Transwell assay. Invasion was increased in HBx-expressing cells relative to control cells, and CAT-1 knockdown inhibited invasion compared to both HBx-expressing cells and siRNA negative control cells (Figure 7).

## DISCUSSION

In this study, we found that Gld2/miR122 levels and CAT-1 activation were inhibited in both HCC tissues and hepatoma cell lines (Figure 1). These observations suggest that dysregulation of Gld2/miR-122/CAT-1 may contribute to the initiation and progression of HCC. Similar changes in Gld2/miR-122/CAT-1 were observed in HepG2.2.15 cells stably expressing HBV. In a previous study, we found that the HBx protein of HBV can reduce miR-122 levels by down-regulating Gld2 in hepatic cells [11]. MiR-122 also reduces CAT-1 expression at the post-transcriptional level [8]. Down-regulation of Gld2/miR-122 and the subsequent activation of CAT-1 may be one mechanism through which HBV affects various cellular activities.

MiR-122 regulates the expression of genes involved in cell proliferation and apoptosis and tumor formation, migration, and invasion [9]. MiR-122 levels are low in certain HCC tissue types and are correlated with poor prognosis, while overexpression of miR-122 inhibits tumor cell growth; animal experiments also indicate that miR-122 acts as a tumor suppressor during hepato-carcinogenesis [10, 13]. Moreover, miR-122 levels are lower than normal level in HBV-associated HCC, but not in liver cancers associated with HCV infection [18]. Together, these finding suggest that HBV infection may contribute to reductions in miR-122 levels in HBV-associated HCC tissues.

HBx reduced miR-122 levels in hepatic cells by inhibiting Gld2 expression, which stabilizes miR-122, in our previous study [11]. Two other studies have confirmed this effect of HBV infection on miR-122 expression in hepatic cells [19, 20]. Here, we found that Gld2 protein levels are reduced in HCC tissues relative to paired noncancerous tissues (Figure 1E). This reduction may contribute to the loss of miR-122 in HCC. Given that miR-122 acts as a tumor suppressor gene, HBV-induced inhibition of the Gld2/miR-122 pathway may also contribute to the formation and progression of HCC. In colorectal cancer cells, down-regulation of miR-122 induced CAT-1 overexpression [21]. However, the effects of miR-122/CAT-1 on HCC cells have not been studied.

Cationic amino acids supplied by CAT proteins feed into protein synthesis and other enzymatic reactions. These reactions include the synthesis of NO from arginine and the synthesis of polyamines, proline, and glutamine from ornithine. An increasing number of studies suggest that CAT-mediated transport is an important regulator of these processes [22, 23]. Although its levels vary, CAT-1 is expressed almost ubiquitously, but it is not expressed in the normal adult liver (Figure 1). However, we found here that CAT-1 proteins were overexpressed in HCC. In addition, reduced miR-122 levels may contribute to this aberrant CAT-1 expression. A previous study showed that over-expression of CAT-1 in HCC cells increased intracellular arginine levels and contributed to hepatocytogenesis, while knockdown of CAT-1 and miR122 together prevented an increase in intracellular NO; these results indicated that reduced miR-122 were a result of increased CAT-1 levels [24]. Our previous *in vitro* experiment demonstrated that endogenous CAT-1 mRNA expression in Huh7 cells was regulated, likely post-transcriptionally, by miR-122 [25]. Previous studies have shown that sustained low CAT-1 activity is necessary in liver cells to avoid hydrolysis of plasma arginine by arginase, which is highly expressed in liver tissues. Increased CAT-1 expression under certain conditions (e.g., when urea cycle enzymes are down-regulated or during liver regeneration) likely helps sustain hepatocellular protein synthesis [23]. CAT-1 can be up-regulated by several factors in the tumor microenvironment, including polyamines, pathologic stress, signals for rapid division, and pro-inflammatory cytokines [26–28]. Regardless, the role of CAT-1 in HCC remains elusive. Our results indicate that CAT-1 overexpression promotes, while CAT-1 knockdown inhibits, HCC proliferation (Figure 2). CAT-1 overexpression also markedly inhibited, while CAT-1 knockdown increased, cell apoptosis (Figure 3). Likewise, CAT-1 knockdown in HCC cells notably decreased cell invasive ability, while ectopic expression of CAT-1 in an HCC cell line with low endogenous expression substantially increased cell invasion (Figure 4).

CAT-1 protein appears to play an important role in intracellular compartmentalization and the channeling of arginine to distinct metabolic pathways [23]. Intracellular arginine is one of the most important amino acids for activation of the mechanistic target of rapamycin (mTOR), particularly the mTORC1 signaling pathway that promotes tumorigenesis, cell survival, and proliferation [21]. CAT-1 also plays a role in the arginine uptake and survival in breast cancer cells [29]. A recent study revealed that acute myeloid leukemia (AML) blasts constitutively express the arginine transporters CAT-1 and CAT2B and that the proliferation of AML blasts depends on arginine [30]. CAT-1 is a novel intercellular endothelial cell adhesion molecule (CAM) which is localized to cell-cell adhesive junctions, similar to the classic vascular endothelial CAM cadherin [31]. We therefore hypothesized that CAT-1 may play a carcinogenic role in HCC by increasing arginine metabolism, but additional research is needed to examine this possibility.

HBx has been implicated in the pathogenesis of HBV-induced HCC [7, 29]. Here, we demonstrated that HBx up-regulates CAT-1 by down-regulating the Gld2/miR-122 pathway (Figure 5), and that CAT-1 knockdown can abrogate HBx-induced increases in proliferation and invasion in HCC cells (Figure 6, Figure 7).

Together, our results suggest that CAT-1 overexpression promotes invasion in HCC cells. Moreover, HBx contributes to the inhibition of the Gld2/miR-122 pathway and subsequent overexpression of CAT-1, thereby increasing cell proliferation and invasion in HBV-related HCC. Therefore, therapies that inhibit CAT-1 might be highly beneficial in the treatment of HCC, and controlling arginine availability and metabolism may be a useful therapeutic approach.

## MATERIALS AND METHODS

### Human tissue samples

Thirty pairs of hepatocellular carcinoma and noncancerous liver tissues were obtained from patients with HBV-related HCC who had undergone hepatectomy and three normal liver tissues who had excluded HCC through pathological examination at The Second Xiangya Hospital. Total RNA and protein were isolated from the tissues using TRIzol Reagent (Invitrogen, USA) and cell culture lysis reagent (Promega), respectively, in a standard grinding bowl pre-cooled with liquid nitrogen. The use of tissues for this study was approved by the Ethics Committee of The Second Xiangya Hospital.

### Plasmid construction

The expression plasmids for miR-122 (pmiR-122) and HBx (pHBx) protein were constructed previously in our laboratory; pHBV1.3 was provided by Doctor Songdong Meng from the Chinese Academy of Sciences (CAS). The CAT-1-expression plasmid (pCAT-1) was cloned from human cDNA (the reverse transcription product from human hepatic cell line QSG7701) using 5'-CGAAGCTTATGGGGTGCAAAGTCCTGCT-3' (sense) and 5'-CGTCTAGATCACTTGCACTGGTCCAAGT-3' (antisense) primers, and pcDNA3.1 (pcDNA) was used as the expression vector.

We designed siRNA targeting HBx and CAT-1 mRNAs according to the GenScript siRNA Target Finder (https://www.genscript.com/ssl-bin/app/rnai). The sense and antisense oligonucleotides that served as the templates for generating the siRNAs were sub-cloned into the pRNAT-U6.1/Neovector with the U6-RNA promoter between the HindIII and BamHI restriction sites. The final sequences selected for HBx siRNA-expressing plasmids (psiHBx) were 5'-TTCACCT CTGCACGTTGCA-3' (sense) and 5'-TGCAACGTGCAGAGGTGAA-3'(antisense). The CAT-1 siRNA target sequences were as follows: siCAT1-1: 5'-GATCCCGCATTTCAACCAGCCTTATATTCAAGAGATATAAGGCTGGTTGAAATGTTTTTTCCAAA-3' (sense) and 5'-AGCTTTTGGAAAAAACATTTCAACCAGCCTTATATCTCTTGAATATAAGGCTGGTTGAAATGCGG-3' (antisense).

The CAT-1 mRNA 3'-UTR luciferase reporter plasmid was cloned by PCR from human cDNA using 5'-CGTTCTAGACGCACAGCCCC GCCC-3' (sense) and 5'-GGGGGCCGGCCTCCTGAAGTAGACTC-3' (antisense) primers and the pmirGLO Dual-Luciferase miRNA Target Expression Vector (Promega). All plasmids were validated by sequencing.

### Cell culture and transfection

QSG7701, L02, Huh7, HepG2, HepG2.2.15, and Hep3B cell lines (purchased from the cell bank of Xiangya Central Experiment Laboratory, China) were cultured in Dulbecco's Modified Eagle’s medium (HyClone) supplemented with 10% fetal bovine serum (FBS; Gibco) and 1% penicillin/streptomycin (Gibco). The cells lines were maintained in a humidified incubator at 37°C with 5% CO_2_. About 24 h prior to transfection, the cells were seeded into 6-well plates in antibiotic-free growth medium at a density of 3×10^5^ cells/well. After reaching 80% confluence, the cells were transfected with vectors using Lipofectamine 2000 (Invitrogen, USA) according to the manufacturer’s instructions.

### Reverse transcription quantitative real-time polymerase chain reaction (RT–qPCR)

Total cellular RNA was isolated using TRIzol Reagent (Invitrogen, USA). Complementary DNA (cDNA) was synthesized from total RNA (both mRNA and miRNA) using a TaKaRa One Step PrimeScript^®^ miRNA cDNA Synthesis Kit (Perfect Real Time) according to the manufacturer’s instructions. To quantify the target mRNA or miRNAs, we performed qPCR with a ABI 7500 Real-Time PCR System and Takara SYBR_Premix Ex TaqTM II (Perfect Real Time) according to the manufacturer’s instructions. The forward primers for each target mRNA or miRNA were as follows: β-actin, 5'-CCAACTGGGACGACAT-3' (sense) and 5'-AGCCTGGATAGCAACG-3'(antisense); CAT-1, 5'-TGCCATTGTCATCTCC-3' (sense) and 5'-TCGCTACGCTTGAAGTA-3' (antisense); U6, 5'-CGCTTCGG CAGCACATATAC-3' (sense) and universal primers provided in the TaKaRa Kit (antisense); miR-122, 5'- TCGCCTGGAGTGTGACAATGG- 3' (sense) and universal primers provided in TaKaRa Kit (antisense). MiRNA and mRNA expression were measured via the Ct (cycle threshold) method. The ΔΔCt method for relative gene expression quantification was used to determine miRNA (or mRNA) expression. ΔCt was calculated by subtracting the Ct of U6 (for miRNA) or β-actin (for mRNA) RNA from the Ct of the miRNA or mRNA of interest. All results were calculated using the 2^-ΔΔCt^ method.

### Western blot analysis

Cells were harvested and lysed in 500 μL of cell culture lysis reagent (Promega) according to the protocol supplied by the manufacturer. The protein concentration of each sample was determined using the BCA™ Protein Assay Kit (Pierce). Standard Western blot procedures were used. The primary antibodies were mouse monoclonal anti-HBx (Santa Cruz, sc-17493), goat polyclonal anti-Gld2 (Santa Cruz, sc-168897), goat polyclonal anti-CAT-1 (Santa Cruz, sc-33087), and mouse monoclonal anti-β-actin (ZSGB-Bio, TA-09). The secondary antibodies used were goat anti-mouse IgG-HRP (Santa Cruz, sc-2005) and donkey anti-goat (Santa Cruz, sc-2020). Antibody/antigen complexes were detected with SuperSignal West Pico Chemiluminescent Substrate (Thermo).

### Immunohistochemistry (IHC)

Thirty pairs of hepatocellular carcinoma and noncancerous liver tissues were fixed in formalin, embedded in paraffin, and cut into individual sections with a thickness of 4 μm. These sections were pre-treated, dewaxed in xylene, and hydrated prior to antigen retrieval. After inhibition of endogenous peroxidase, the sections were incubated overnight at 4°C with a goat polyclonal antibody against human Gld2 (Santa Cruz, sc-168897) and CAT-1 (Santa Cruz, sc-33087). After thorough washing with phosphate-buffered saline (PBS), corresponding secondary antibodies (donkey anti-goat, Santa Cruz, sc-2020) were applied and incubated at room temperature for 30 min. Reaction products were visualized by incubation with 3,3'-diaminobenzidine (DAB) and then counterstained with hematoxylin. Negative controls were obtained by substituting the primary antibody with an isotype-matched irrelevant antibody.

### Dual-luciferase assay

The expression vectors for these genes (or the corresponding null vectors) and the CAT-1 mRNA 3'-UTR dual-luciferase reporter plasmids were mixed (4:1) and co-transfected into cells cultured in 6-well plates. The cells were split, and FL/RL activity was measured 48 h post-transfection with the Dual-Luciferase Assay Kit (Promega).

### Colony formation assay

HepG2 cells were seeded in 6-well plates at a low density (1,000 cells per well) and then cultured for 6 days. The plates were then washed with PBS, fixed with 10% methanol, and stained with Azure eosin methylene blue (Giemsa stain). Images of each well were examined and individual clone types were identified.

### MTT assay

Cell proliferation was examined using the 3-(4, 5-dimethylthiazol-2-yl)-2, 5-diphenyl tetrazolium bromide (MTT) assay. HepG2, HepG2.2.1.5, Huh7, Hep3B cells were seeded in 96-well plate chambers at a concentration of 5000 cells/well. After 48 h, the cells were incubated with 0.5 mg/mL MTT for another 4 h. After 100 μL of dimethyl sulfoxide was added, absorbance was measured at 492 nm using a Wellscan MK3 (ELISA reader).

### Cell apoptosis analysis

The apoptosis assay was conducted using the Annexin V-7AAD Apoptosis Detection Kit (Keygen Biotech) according to manufacturer’s instructions. The cells were then analyzed using FACS flow cytometry (BD Biosciences Inc.)

### Transwell invasion assay

The invasion assay was performed using a Transwell chamber (PIEP12R48, Millipore, USA). HepG2 cells were seeded into the upper chamber with serum-free medium (2.5×10^4^ cells); the bottom of the chamber contained HyClone Medium with 5% FBS. The chamber was coated with Matrigel. After 20 hours of cell invasion, the cells were fixed and stained with crystal violet, photographed under an optical microscope (Hanrong Company, Shanghai), and counted.

### Data analysis

All experiments were replicated independently 3 times, and triplicate wells were transfected for each experiment. Data were analyzed using a two-tailed Student’s *t*-test with pooled variance. *P*<0.05 was considered statistically significant. All statistical analyses were performed in SPSS 16.0.
